# Cottage Hospital Practice

**Published:** 1907-05-25

**Authors:** 


### EDITOR'S LETTER-BOX.

[Our Correspondents are reminded that prolixity is a great bar tc publication. and that brevity of style and conciseness ot statement greatly facilitate early insertion.]

### COTTAGE HOSPITAL PRACTICE.

BILL BERRY writes :—Can you or any of your many readers kindly assist my committee with practical advice on the following matter of hospital administration?

Our cottage hospital is used for the general objects and purposes of such an institution, and is managed by a committee. Patients of the usual kind are admitted, with or without weekly payment acordingly as the recommending committee-man or the committee themselves consider they can afford.

The medical staff of the hospital consists of those practitioners who live within a ten-mile radius, and the one local doctor, who resides in the village in which the hospital is situated. The former are not paid, but the latter receives a fixed remuneration for the performance of certain duties specified in the by-laws. The patients can be attended by their own doctors if on the staff.

The majority of cases are sent in by the local practitioner.

A bad accident happens in the immediate neighbourhood. The victim, who is in a fairly well-to-do position, is carried into the hospital late in the evening. His regular medical attendant lives some miles away, but is on the staff, and is sent for by the patient's friends. He comes over, performs

The committee meet in the meantime, consider the case, successfully until it is discharged off the books.

The committee meet in the meantime, consider the case. and impose a weekly payment from the patient or his friends.

The patient's doctor, on the termination of the case, sends in to the patient privately his bill for attendance, after expressing to the Secretary of the hospital his intention to charge.

Should the doctor be permitted to charge the patient under the circumstances, or, if not, how can the committee prevent it?

It may be added that the committee do not grudge the doctor his private charges on the patient in this particular case, but consider they were well earned; but their point is rather, Should a patient, who is brought into a charitable institution and had a weekly payment supposed to be commensurate with his means put upon him, be charged by his own doctor, who is an *honorary* member of the hospital's medical staff?

And this seems to lead to some further points, namely: If in one case a patient is permitted, with the knowledge of the committee, to be charged by his medical man attending him, why should not another case be similarly treated, even though the latter may not be so well able to afford payment? If a doctor on the honorary staff attending a patient in the hospital sends him in a bill for attendance without reference to the committee and receives payment, will not this come within the Prevention of Corruption Act, 1906? And, finally, May not the committee, who are simply trustees for carrying out a benevolent trust, who connive at a practice of a member of the medical staff charging a patient privately be sailing close to the wind under the same Act? Scores of somewhat similar cases of emergency admissions must have occurred at cottage hospitals up and down the country, and it would be very interesting and instructive to have the views of those experienced in the details of management of these very useful institutions.

